# Successful CD19 chimeric antigen receptor T-cell therapy following autologous stem cell transplantation in a secondary central nervous system lymphoma patient with COVID-19 infection: a case report and literature review

**DOI:** 10.3389/fonc.2025.1656034

**Published:** 2025-09-05

**Authors:** Xiaoning Wang, Juan Ren, Yuqi Wang, Minna Luo, Jing Li, Pengcheng He

**Affiliations:** Department of Hematology, The First Affiliated Hospital of Xi’an Jiaotong University, Xi’an, China

**Keywords:** diffuse large B-cell lymphoma, secondary central nervous system lymphoma, CD19 chimeric antigen receptor T-cell, autologous stem cell transplantation, COVID-19

## Abstract

This paper reported a 64-year-old, secondary central nervous system lymphoma (sCNSL) patient with COVID-19 infection during CD19 chimeric antigen receptor T-cell (CAR-T) therapy following autologous stem cell transplantation (ASCT). Our findings demonstrate that the combination of ASCT and CAR-T for sCNSL may be feasible, even for SARS-CoV-2-positive immunocompromised lymphoma patients.

## Introduction

Patients with secondary central nervous system lymphoma (sCNSL) have a very poor prognosis and have an urgent need for effective treatment. Only few reports support the use of autologous stem cell transplantation (ASCT) for sCNSL, owing to unsuccessful stem cell harvest and the high incidence of relapse after ASCT. CD19 chimeric antigen receptor T-cell (CAR-T) therapy is a novel treatment strategy for relapsed or refractory B-cell malignancies. Patients with central nervous lymphoma were excluded from most trials due to concerns for cytokine release syndrome (CRS) of the central nervous system (1–3).With the improvement of CAR-T production and complication management, CAR T-cell immunotherapy following ASCT for central nervous system lymphoma has been reported recently. However, very few subjects were included (4, 5).

COVID-19 infection had a dramatic impact on the mortality of patients with hematological malignancies, particularly for patients who underwent ASCT or CAR-T therapy. Mortality rates may escalate up to 40% (6). This raised concerns about initiating ASCT or CAR-T therapy in patients with COVID-19 infection, due to the potential for development of severe pneumonia or acute respiratory distress syndrome. The present study first reports the treatment of ASCT and CAR-T therapy in an sCNSL patient with concurrent COVID-19 infection and makes a literature review. We aimed to provide insights into the therapeutic strategy for sCNSL patients with COVID-19 infection.

## Case report

A 64-year-old man complaining of pain in the upper abdomen was admitted to the Department of Hematology, the First Affiliated Hospital of Xi’an Jiaotong University on 6 February 2023. The patient had no history of specific disease, family disease, or genetic disease. The complete blood count test showed the following results: white blood cell count, 7.68 × 10^9^/L; hemoglobin level, 140 g/L; and platelet count, 445 × 10^9^/L. Additional laboratory tests showed that the serum lactic dehydrogenase level was 539 U/L (upper limit of normal: 250 U/L). Positron emission tomography/computed tomography (PET/CT) revealed multiple enlarged lymph nodes in the right axillary, chest wall muscle spaces, and retroperitoneum. The patient was diagnosed as having diffuse large B-cell lymphoma (DLBCL) by right axillary lymph node biopsy. The immunohistochemistry showed CD20(+), CD19(+), CD3(−), CD5(−), Bcl2(+80%), C-myc(+70%), CD10(−), Bcl6(+), Mum1(+), CyclinD1 (−), CD30(−), P53(+80%), and Ki67(+80%) and that the patient had a 46, XY(20) karyotype.

The patient received two courses of R-CHOP (rituximab 700 mg on day 0, vindesine 4 mg on day 1, ifosfamide 1.5 g on day 1, doxorubicin liposome 40 mg on day 1, 20 mg on day 2, and prednisone 60 mg on days 1–5). Twenty-one days after the last round of therapy, PET/CT showed that the lymph nodes of the right axillary, chest wall muscle spaces, and retroperitoneum were enlarged. Therefore, the patient was given six cycles of R-Pola-Gemox therapy (rituximab 700 mg on day 1, pola 131 mg on day 1, gemcitabine 1,870 mg on day 2, and oxaliplatin 187 mg on day 2) and two cycles of rituximab 700 mg and gemcitabine 1,870 mg and then chemotherapy was stopped. After four cycles of R-Pola-Gemox therapy, the status of the disease was complete metabolic remission evaluated by PET/CT. Nine months after the treatment ended, the patient had a headache. Cranial magnetic resonance imaging (MRI) showed a left frontal lobe mass with peripheral edema. PET/CT showed new soft tissue nodules in the deep left frontal lobe and basal ganglia area with increased glucose metabolism, which indicated relapse of the disease. Robot-assisted stereotactic biopsy under general anesthesia was done and the pathology showed “left frontal” diffuse large B-cell lymphoma (germinal center type). The immunohistochemistry of biopsy showed the following: LCA(+), CD20(+), CD19(+), CD3(−), CD5(−), CyclinD1(−), CD30(−), Bcl-2(−), Bcl-6(40%+), C-myc(10%+), MUM1(+), CD10(+), Ki67 (70%+), P53 (wild type), and ALK-p80(−). The patient received two cycles of rituximab 600 mg on day 0, methotrexate 5,500 mg on day 1, and temozolomide 150 mg on day 3 and 200 mg on days 4–7. Then, lymphocytes were collected, and 1 month later, the peripheral hematopoietic stem cells were harvested. The conditioning regimen for the patient undergoing ASCT was TEAM (thiotepa, etoposide, cytarabine, and melphalan), which included thiotepa (5 mg/kg) on day −7, cytarabine (200 mg/m^2^) every 12 h, etoposide (200 mg/m^2^) on days −6 to −3, and melphalan (140 mg/m^2^) on day −2. During the conditioning, the patient developed a fever (37.8 °C) and nasal congestion on day −4; the COVID-19 polymerase chain reaction (PCR) on the nasal swab sample was positive, which resulted in a negative nasal swab after molnupiravir treatment. Autologous hematopoietic stem cells were infused on day 0 with a mononuclear cell dose of 6.44 × 10^8^/kg and a CD34^+^ cell dose of 6.79 × 10^6^/kg. On day +1, he developed a fever (38 °C) due to agranulocytosis; imipenem cilastatin in combination with caspofungin was thus given but the patient still had a low grade fever. The COVID-19 PCR test on the nasal swab sample was again positive and became negative after 5 days’ treatment of nirmatrelvir/ritonavir. Concurrent medications of nirmatrelvir/ritonavir included levetiracetam, carbopenem, imipenem cilastatin, calcium leucovorin, ursodeoxycholic acid, prostaglandin E1, SMZ-TMP, and acyclovir; dose adjustments of these drugs were not required.

**Figure 1:**
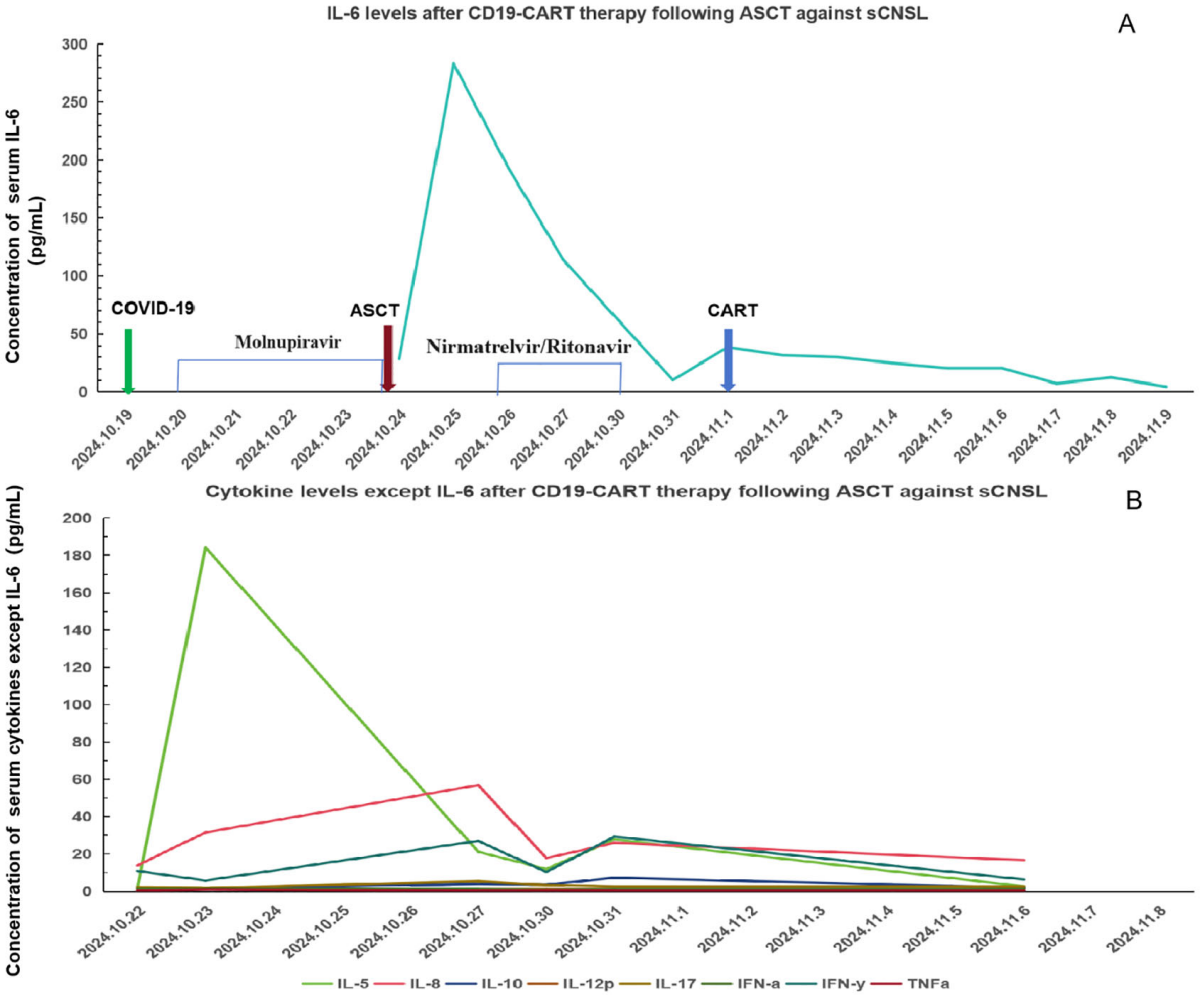
Cytokine levels after CD19-CAR-T therapy following ASCT against sCNSL **(A)** IL-6; levels after CD19-CART therapy following ASCT against sCNSL, peaking on October 25, 2024, and declining after. **(B)** Levels of other cytokines, excluding IL-6, over the same period.

**Figure 2:**
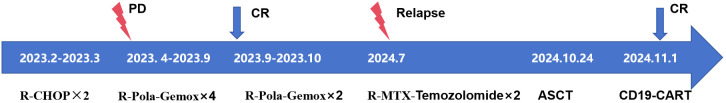
Timeline diagram showing treatment progression.

**Figure 3:**
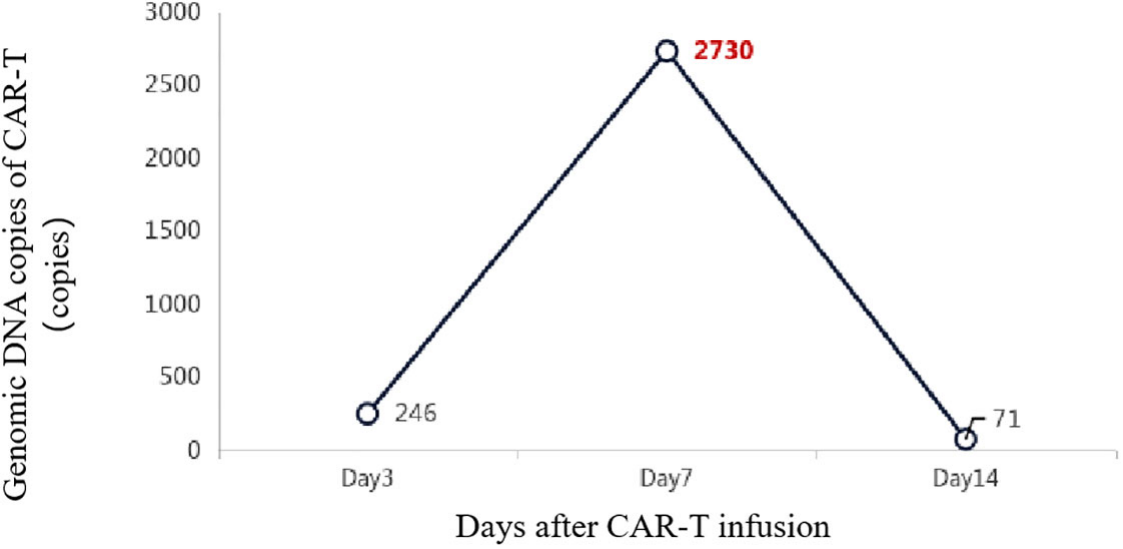
The *in vivo* expansion and persistence of CAR-T cells were monitored by RT-PCR.

**Figure 4:**
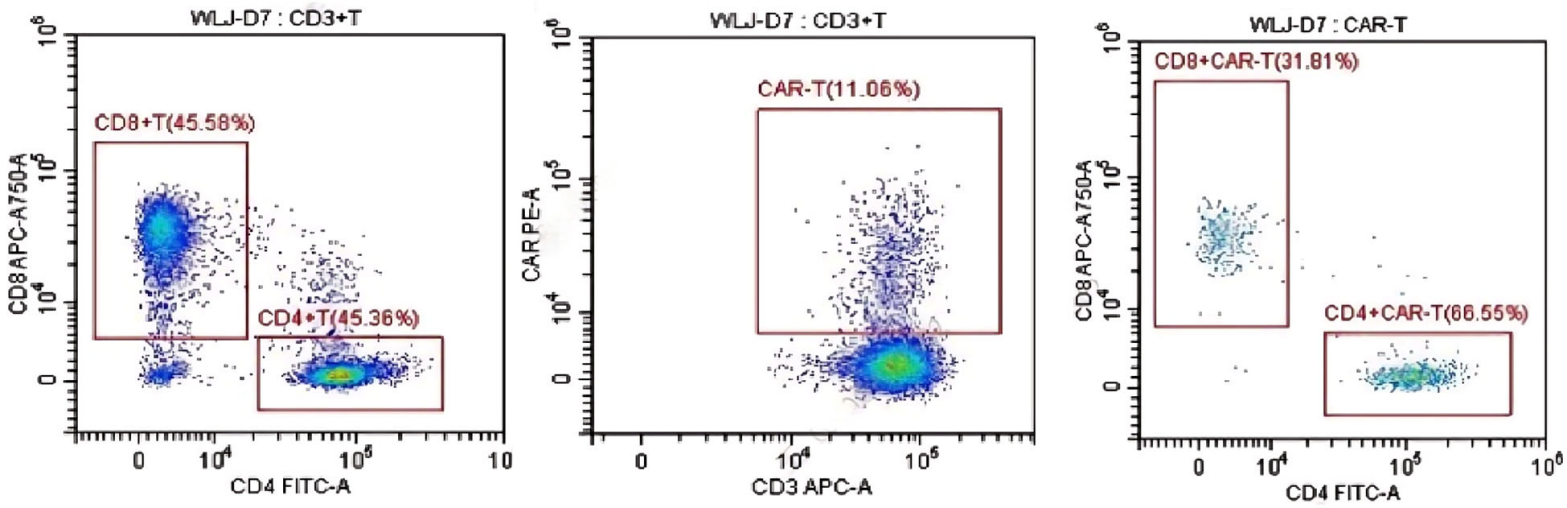
The *in vivo* persistence of CAR-T cells 7 days after infusion was monitored by a flow cytometer.

Additionally, 2 × 10^6^/kg CD19-CAR-T cells were infused 8 days after ASCT. There were no manifestations of CRS and neurotoxicity in this patient. The engraftment times of neutrophil and platelet were 10 days and 10 days after ASCT, respectively. The cytokine levels after CAR-T therapy are shown in **Figure 1**. The patient achieved complete remission and received zanubrutinib maintenance therapy from 3 months after CAR-T therapy and was still in complete remission for 6 months. The follow-up is still ongoing, the blood routine was normal, and the patient had no infection with B-cell aplasia during the follow-up. The treatment regimen of the patient is depicted in **Figure 2**. The *in vivo* expansion and persistence of CAR-T cells were monitored by reverse transcription polymerase chain reaction (RT-PCR) and a flow cytometer (**Figures 3**, **4**). The copies of CAR-T cells reached their first peak 7 days after infusion and then dropped.

## Discussion

Central nervous system involvement in DLBCL accounts for 5%–10% of DLBCL cases and is associated with very poor prognosis, with a median survival of less than 6 months. Traditional treatment approaches included high-dose methotrexate-based chemotherapy combined with whole-brain radiotherapy, but long-term survival rates remained below 30%. ASCT as consolidation therapy following high-dose chemotherapy can improve survival in some patients. However, because of the blood–brain barrier, drug penetration is limited, and central nervous relapse rates after ASCT remained high. Targeted drugs such as Bruton’s tyrosine kinase (BTK) inhibitors, interleukin-1 receptor-associated kinase-4 inhibitors, and immunomodulators including lenalidomide and pomalidomide had also show certain efficacy in central nervous system lymphoma. In order to further improve the efficacy, most of these drugs were combined with other chemotherapy regimens or used for maintenance therapy. In recent years, CD19-CAR-T therapy has demonstrated significant efficacy in relapsed/refractory DLBCL, particularly showing some penetration into central nervous system lesions, offering new hope for DLBCL patients with central nervous system infiltration (7–10). Nevertheless, neurotoxicity following CAR-T therapy [e.g., immune effector cell-associated neurotoxicity syndrome (ICANS)] and the impact of the central nervous system-specific microenvironment required further investigation.

Currently, there is no standard approach for the optimal sequencing of ASCT and CAR-T therapy. Clinical decision-making must consider the patient’s disease status, prior treatment response, and tolerability. Existing research supports two primary strategies: For chemotherapy-sensitive patients eligible for transplantation, ASCT first can maximally reduce tumor burden and enhance the efficacy of subsequent CAR-T therapy. **Table 1** shows several reports about the strategy of ASCT prior to CAR-T therapy. Patients achieving partial remission or with minimal residual disease positive after ASCT may experience prolonged progression-free survival with CAR-T therapy. Additionally, the lymphocyte-depleting effect of ASCT may enhance CAR-T cell expansion and persistence. However, delayed immune recovery after ASCT may increase infection risk, necessitating careful evaluation. For patients with primary resistance or early relapse after ASCT, prioritizing CAR-T therapy may be more appropriate. CAR-T can induce deep remission, followed by ASCT to eliminate residual disease, particularly in high-risk genetic subgroups (e.g., double-hit or triple-hit lymphomas). However, long-term remission rates after CAR-T therapy remained limited.

**Table 1:** Treatment and outcomes from prior trials of ASCT combined with CAR-T cell therapy for central nervous system lymphoma (literature review).

Published online	Numbers of patients	Age (years)	Target	CRS	Response	Survival
Sylvain Choquet, 2024 (11)	14	68 (34–76)	CD19	92%	80%	Median OS, 21.2 months
Xiaoxi Zhou, 2024 (5)	29	42 (23–66)	CD19+CD22	41.4% (20.7% ICANS)	82.75%	2-year OS, 72%
Fei Xue, 2022 (4)	8	42 (32–66)	CD19/CD20/CD22	41% (29% ICANS)	100%	Median OS, 19.3 months
Jiaying Wu, 2021 (12)	13	42 (23–65)	CD19+CD22	23%	84.61%	1-year OS, 82.5%

CRS, cytokine release syndrome. ICANAS, immune effector cell-associated neurotoxicity syndrome; OS, overall survival.

Because of the lymphodepleting chemotherapy, persistent B-cell aplasia, hypogammaglobulinemia, and CRS, patients who received CD19-CAR-T therapy had high risk of infections, especially the viral kind. Early diagnosis and effective antiviral treatment were crucial for reducing mortality. In the post-COVID-19 era, the impact of COVID-19 on CAR-T therapy needed to be taken seriously. It was recommended that nucleic acid testing for COVID-19 was done before CAR-T therapy. For hospitalized COVID-19 patients, concurrent antiviral therapy with corticosteroids may decrease the risk of CRS while reducing viral load (13). The utilization of IL-6 inhibitors and JAK inhibitors in severe COVID-19 cases required careful consideration, especially for lymphoma patients with B-cell aplasia. Controlled trials had demonstrated that early utilization of antiviral drugs such as molnupiravir and nirmatrelvir/ritonavir may significantly reduce mortality. Tixagevimab/cilgavimab was recommended for prophylaxis during the COVID-19 pandemic, particularly for patients within 1 year of CAR-T therapy (13).

This paper is the first to report successful CD19-CAR-T therapy following ASCT in an sCNSL patient with COVID-19 infection. It may indicate the feasibility of ASCT and CAR-T therapy for immunocompromised patients with COVID-19 infection.

For immunocompromised patients with COVID-19 infection during the treatment, they present distinct challenges, characterized by prolonged viral shedding, severe infections being more likely in these patients, and the release of inflammatory factors exacerbating CRS. This was a complex decision-making process for both physicians and the patient. The patient positive for COVID-19 did not pursue CAR-T therapy, especially considering the absence of pulmonary involvement. Early detection and intervention, comprehensive management, and collaborative decision-making were important for achieving positive outcomes for patients who face dual challenges of immunosuppression and COVID-19 infection (14). For this case, the patient did not have COVID-19 infection before admission, and at that time, there was a COVID-19 epidemic in the hospital; thus, the patient becoming COVID-19 positive may be considered as nosocomial infection. When patients develop a fever, they were screened for COVID-19 and received antiviral and anti-infection supportive treatment, so that severe pneumonia and CRS, especially ICANS, may be prevented. This also reminded us about the importance of early diagnosis and treatment; in addition, we needed to enhance the prevention and control of infection during the epidemic period.

Maintenance therapy after sequential treatment may further delay relapse, particularly in sCNSL patients. Potential options include BTK inhibitors (e.g., zanubrutinib and ibrutinib). These agents can penetrate the blood–brain barrier and inhibit B-cell receptor signaling, reducing central nervous system relapse. Immunomodulatory drugs (e.g., lenalidomide) enhance T-cell function and remodel the anti-tumor immune microenvironment. PD-1/PD-L1 inhibitors have shown activity in some central nervous system lymphomas but require caution due to immune-related adverse effects. The indications, duration, and optimal combinations for maintenance therapy still require prospective validation (15–17). The patient in this case report began maintenance treatment with BTK inhibitors approximately 3 months after treatment, and the follow-up time is still short; thus, we need to continue monitoring the changes in efficacy.

## Data Availability

The raw data supporting the conclusions of this article will be made available by the authors, without undue reservation.
